# Blood-Based Proteomic Biomarkers of Alzheimer’s Disease Pathology

**DOI:** 10.3389/fneur.2015.00236

**Published:** 2015-11-16

**Authors:** Alison L. Baird, Sarah Westwood, Simon Lovestone

**Affiliations:** ^1^Department of Psychiatry, University of Oxford, Oxford, UK

**Keywords:** Alzheimer’s disease, biomarkers, blood, proteomics, dementia

## Abstract

The complexity of Alzheimer’s disease (AD) and its long prodromal phase poses challenges for early diagnosis and yet allows for the possibility of the development of disease modifying treatments for secondary prevention. It is, therefore, of importance to develop biomarkers, in particular, in the preclinical or early phases that reflect the pathological characteristics of the disease and, moreover, could be of utility in triaging subjects for preventative therapeutic clinical trials. Much research has sought biomarkers for diagnostic purposes by comparing affected people to unaffected controls. However, given that AD pathology precedes disease onset, a pathology endophenotype design for biomarker discovery creates the opportunity for detection of much earlier markers of disease. Blood-based biomarkers potentially provide a minimally invasive option for this purpose and research in the field has adopted various “omics” approaches in order to achieve this. This review will, therefore, examine the current literature regarding blood-based proteomic biomarkers of AD and its associated pathology.

## Introduction

Dementia is now a huge public health priority, with 115.4 million people worldwide estimated to be living with dementia by 2050 (1). These numbers are not only alarming on an individual level, but they are also unsustainable for our economy. Dementia costs the global economy US$604 billion, and like prevalence rates this figure is also set to increase with an 85% rise in costs estimated by the year 2030 (2).

The most common form of dementia is Alzheimer’s disease (AD), comprising approximately 50–70% of the elderly dementia population. AD is characterized by multiple cognitive deficits, which cause significant impairment to social or occupational functioning. The disease typically has a gradual onset followed by continuing cognitive decline, with a mean duration of approximately 8.5 years from the onset of clinical symptoms to the death of the patient (3).

Most clinical trials of potential therapeutic disease-modifying agents have involved individuals with clinically manifest dementia and have been relatively unsuccessful to date. Earlier stages of the disease are now being targeted, posing a challenge as it is difficult to detect individuals at this stage of AD; brain pathology is developing silently and cognitive symptoms if detectable are subtle. The underlying neuropathology characteristic of AD precedes symptom onset by many years, with the accumulation of amyloid-beta (Aβ) plaques believed to occur 15–20 years in advance of clinical manifestation of the disease (4), followed by the aggregation of abnormally phosphorylated tau in neurofibrillary tangles. A biological marker (biomarker) of these pathologic processes could serve as an indicator of disease presence, pathology, and progression. Moreover, they could have great utility in drug development and clinical trials, in particular for use in patient stratification and cohort enrichment.

In this review, we will discuss various studies that have utilized proteomic-based approaches to discover blood-based biomarkers for early and ideally preclinical detection of AD pathological processes and their use in clinical trials. We performed literature searches on PubMed^1^ using the search terms detailed in Table 1. The literature included for review was supplemented with other known applicable papers that were not identified in the searches.

**Table 1:** **Search terms used for PubMed-based literature searches. Publications were filtered to include only studies in human species**.

Search	Search terms
Plasma Aβ and Tau as biomarkers of AD	[alzheimer*(Title/Abstract) OR dementia(Title/Abstract) AND AD(Title/Abstract)] AND [blood(Title/Abstract) OR plasma(Title/Abstract) OR serum(Title/Abstract)] AND [proteomic*(Title/Abstract) OR proteome(Title/Abstract) OR protein(Title/Abstract) OR proteins(Title/Abstract)] AND [biomarker*(Title/Abstract) OR marker*(Title/Abstract)] AND [beta-amyloid(Title/Abstract) OR amyloid beta(Title/Abstract) OR abeta(Title/Abstract) OR tau(Title/Abstract)]
Plasma biomarkers of AD (case–control studies)	[alzheimer*(Title/Abstract) OR dementia(Title/Abstract) AND AD(Title/Abstract)] AND [blood(Title/Abstract) OR plasma(Title/Abstract) OR serum(Title/Abstract)] AND [proteomic*(Title/Abstract) OR proteome(Title/Abstract) OR protein(Title/Abstract) OR proteins(Title/Abstract)] AND [biomarker*(Title/Abstract) OR marker*(Title/Abstract)] AND [diagnos*(Title/Abstract) OR prognos*(Title/Abstract) OR progression(Title/Abstract)]
Plasma biomarkers of brain atrophy	[alzheimer*(Title/Abstract) OR dementia(Title/Abstract) AND AD(Title/Abstract)] AND [blood(Title/Abstract) OR plasma(Title/Abstract) OR serum(Title/Abstract)] AND [proteomic*(Title/Abstract) OR proteome(Title/Abstract) OR protein(Title/Abstract) OR proteins(Title/Abstract)] AND [biomarker*(Title/Abstract) OR marker*(Title/Abstract)] AND [atrophy(Title/Abstract) OR brain volume(Title/Abstract) OR sMRI(Title/Abstract) OR structural magnetic resonance imaging(Title/Abstract) OR structural MRI(Title/Abstract)]
Plasma biomarkers of cognitive decline	[alzheimer*(Title/Abstract) OR dementia(Title/Abstract) AND AD(Title/Abstract)] AND [blood(Title/Abstract) OR plasma(Title/Abstract) OR serum(Title/Abstract)] AND [proteomic*(Title/Abstract) OR proteome(Title/Abstract) OR protein(Title/Abstract) OR proteins(Title/Abstract)] AND [biomarker*(Title/Abstract) OR marker*(Title/Abstract)] AND [cognitive decline(Title/Abstract) OR cognition(Title/Abstract) OR MMSE(Title/Abstract) OR ADAS(Title/Abstract) OR CDR(Title/Abstract)]
Plasma biomarkers of PET amyloid	[alzheimer*(Title/Abstract) OR dementia(Title/Abstract) AND AD(Title/Abstract)] AND [blood(Title/Abstract) OR plasma(Title/Abstract) OR serum(Title/Abstract)] AND [proteomic*(Title/Abstract) OR proteome(Title/Abstract) OR protein(Title/Abstract) OR proteins(Title/Abstract)] AND [biomarker*(Title/Abstract) OR marker*(Title/Abstract)] AND [pib(Title/Abstract) OR Pittsburgh compound b(Title/Abstract) OR florbetapir(Title/Abstract) OR flutemetamol(Title/Abstract) OR florbetaben(Title/Abstract) OR amyloid PET(Title/Abstract) OR brain amyloid(Title/Abstract)]

## Biomarkers for AD

**Figure 1:**
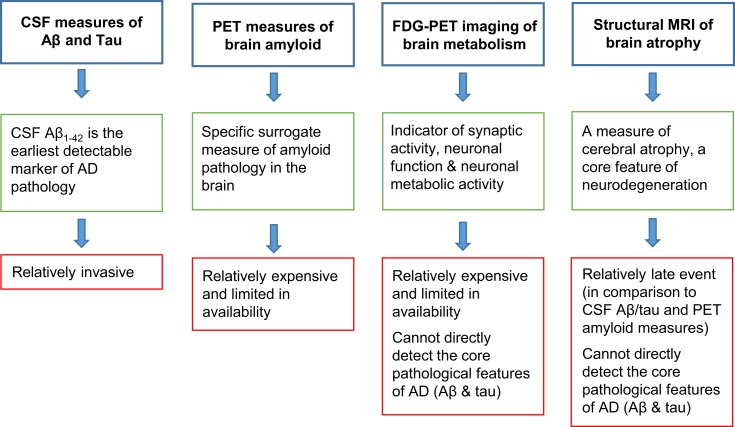
**Advantages (green boxes) and disadvantages (red boxes) of the biomarkers that are currently most widely used in clinical trials**.

Today, the biomarkers used most extensively in clinical trials for dementia and to some extent in clinical practice are structural magnetic resonance imaging (MRI), molecular imaging of amyloid deposition using positron emission tomography (PET), imaging of metabolism using fluoro-deoxy-d-glucose (FDG)-PET, and cerebrospinal fluid (CSF) measures of Aβ and tau. However, structural changes measured using MRI are most likely relatively late events in the disease course and PET imaging is relatively expensive and limited in availability. Moreover, structural MRI and FDG-PET are not direct measures of the core pathological hallmarks of AD (Aβ and tau) and may, therefore, be relatively non-specific for AD in some cases (5) (Figure 1).

The most well-characterized and validated tissue fluid molecular-based biomarker for AD is the decrease in Aβ and increase in tau and phospho-tau (pTau) observed in the CSF of people with AD, with a number of studies documenting discrimination of AD patients from healthy controls with good sensitivity and specificity, as reviewed by others (6). However, the clinical utility of this biomarker is limited by the relatively invasive nature of obtaining CSF (lumbar puncture), particularly from elderly individuals. This may limit its use in longitudinal studies or for clinical progression monitoring, for which repeated CSF measures would be required. Also, attention needs to be paid to standardization of measurement of these biomarkers, given that large inter-laboratory variation in the concentrations measured of these biomarkers are observed (7).

In a revised model of the temporal relationship between key biomarkers of AD pathology, Jack et al. (8) suggested that changes in CSF Aβ_1–42_ are the earliest detectable biomarker of AD pathology, followed by the PET detection of changes in brain amyloid, changes in CSF tau levels, and finally MRI-based detection of hippocampal atrophy and FDG-PET measures of brain glucose metabolism, all of which occur prior to the emergence of clinical symptoms of the disease (8). This hypothesis is corroborated by the recent findings of a non-linear association between CSF Aβ_1–42_ and florbetapir F-18 PET imaging of brain amyloid load at the extreme ends of the clinical scale, while strongest association is seen at the mid-range of clinically measured disease severity (9). These findings would suggest that the two measures could reflect different aspects of AD Aβ pathology, with PET ligands having poor affinity for diffuse plaques, which develop early. At this stage, the plaques retain Aβ and, hence, CSF measures of Aβ may be more sensitive than PET earlier on in the disease (9). It is, therefore, of importance to consider the sensitivity of these biomarkers in relation to the staging of disease when designing biomarker studies.

## Blood-Based Biomarkers

The minimally invasive and potentially inexpensive nature of tests using blood-based proteomic biomarkers make these approaches practical to implement, allowing for repeated sampling in large cohorts, and, therefore, might have significant advantages over other biomarker modalities. However, the use of blood as a matrix for measurement of biomarkers has the inherent disadvantage of its complex composition and subsequently poses technical difficulties for biomarker detection.

The most challenging of many obstacles to developing blood-based biomarkers is the massive dynamic range of proteins in blood, spanning up to 12 orders of magnitude (10). Furthermore, changes within the blood are often very small and reflect a wide range of both peripheral and central processes and, therefore, pinpointing AD-specific changes can be challenging. Separated by the blood–brain barrier the relationship between analytes found in the blood and changes in the brain is still uncertain. However, with aging and in AD, the blood–brain barrier is disrupted resulting in increased permeability, and this is thought to be a relatively early event in the aging brain, worsening with increased cognitive impairment (11). Blood–brain barrier disruption should only strengthen the relationship between blood and brain, and as an early event this would support the possibility of detecting protein-based markers related to AD at the early stages of disease. Nevertheless, concentrations of most known potential biomarkers are considerably lower in the blood than reported in CSF. For example, Aβ peptide concentration is 100-fold lower in blood than in CSF (12). Additionally, highly abundant plasma proteins such as albumin and IgG may mask the presence of less abundant proteins that may serve as potential biomarkers.

## Techniques for Blood-Based Biomarker Discovery

The complexity of blood as a source of biomarkers is reflected in the limitations of various proteomic techniques that have been employed to investigate blood-based biomarkers for AD. In the following section, we will provide a brief overview of some of the tools available for proteomic biomarker discovery in blood, including mass spectrometry (MS), immunocapture, and aptamer-based techniques. Each of these approaches has their advantages and disadvantages and to date studies have combined a number of these approaches in the discovery pipeline for identifying protein biomarkers related to AD.

### Mass Spectrometry-Based Assays

For discovery-level proteomics, a key attribute required of the technique used is the ability to measure multiple targets simultaneously in a multiplexing manner. MS-based approaches have been widely used in this way and possess the inherent advantage of there being no requirement for prior knowledge of the proteins being identified, hence, allowing for unbiased hypothesis-free biomarker discovery. Moreover, to facilitate multiplexing capabilities of MS-based protein quantification, approaches for labeling peptides or intact proteins have been developed, for example, the use of isobaric tags (13). This allows for the pooling of labeled samples for subsequent MS analysis, hence, increasing throughput. However, disadvantages of labeling may include increased complexity in sample preparation compared to label-free approaches. Furthermore, quantitative information can only be provided for peptides that contain the labeled amino acid, limiting the quantitative coverage of the sample to the labeled peptides alone.

However, the huge abundance of a select few proteins in plasma and serum limits the detection of lower molecular weight proteins by MS. In plasma and serum, albumin and the immunoglubulins (IgG, IgA, IgM, and IgD) represent 75% of the total protein weight, and 99% of these samples are constituted by only 22 different protein species (14). Fractionation of the sample is one of the approaches that can be taken to reduce this sample complexity (15). Immunoaffinity-based depletion of the most abundant proteins is another approach that can be used to improve the detection of lower molecular weight proteins and the use of several different immunoaffinity depletion reagents have been documented (16–18). However, a disadvantage of depletion is the potential removal of lower molecular weight proteins, in addition to the high molecular weight targets for depletion. This is mainly an issue due to the binding nature of the protein targets for depletion, such as albumin, and, therefore, by removal of albumin, inadvertently albumin-bound lower molecular weight proteins may also be removed.

### Immunocapture-Based Assays

The gold standard for soluble protein quantification is ELISA. However, with the increasing need to measure multiple protein targets, with limited sample availability, multiplexing approaches for targeted and hypothesis-driven biomarker discovery are now increasingly being used. Two of the most widely used immunocapture-based multiplexing systems for this purpose are mesoscale discovery (MSD) and the Luminex xMAP technology.

Both MSD and Luminex xMAP technologies are similar to the “sandwich” ELISA in priniciple. However, in an MSD assay, the capture antibodies are coated on specific spot regions at the base of the wells of a microtiter plate. Capture antibodies for different targets can be coated on each of the different spots, thus, allowing for multiple protein targets to be captured simultaneously in a single sample. Electrochemiluminescence (SULFO-TAG) labels are then bound to the detection antibodies and upon electrical stimulation the SULFO-TAG labels emit light, which is used to quantify the amount of target protein present. Luminex xMAP assays, in contrast, use microsphere-based technology, which involves coating of the capture antibody to microspheres “beads” in suspension, and fluorescently labeled detection antibodies for detection and quantification. In this way, multiple beads may be coated with multiple capture antibodies for multiplexing protein measurements in a single sample. Whichever approach to multiplexed affinity capture is used, the method is dependent on the quality, binding characteristics, and batch stability of the primary (and indeed secondary) antibodies used.

Given the targeted nature of immunocapture-based assays, these approaches are not necessarily suitable for unbiased hypothesis-free approaches for biomarker discovery. Furthermore, protein quantification by immunocapture methods will be epitope specific, and the quantitative values obtained will relate to the region of the protein recognized by the antibodies used within the assay. This is an important property to note when using immunocapture-based methods for replication of findings that may have been discovered on a different methodological platform, such as MS. Where failure to technically replicate data between platforms is observed, it could be due to differences in the region of the protein being recognized by the different assays. Platform and assay differences in protein quantification are, therefore, important points to consider when designing the pipeline for biomarker discovery and development.

### Aptamer-Based Assays

Aptamer-based approaches also provide another approach for relative quantification of multiple proteins in a multiplexing manner. Aptamers are single-stranded oligonucleotides, which recognize and bind target proteins with high affinity and specificity. Using this technology, the protein signal is effectively transformed to a nucleotide signal for subsequent microarray-based quantification of the relative fluorescence levels. An example of this approach is the panel that Somalogic has developed, which measures over 1300 analytes in a single sample^2^. Advantages of this technology are clearly the large and unrivaled number of protein targets that can be quantified simultaneously in a single sample, making this platform ideal for extensive proteomic analysis in samples of limited availability. However, proteins quantified in this way are limited to those for which aptamers have been designed just as immunoaffinity approaches are limited by antibody availability.

Each of the proteomic techniques described here have inherent advantages and disadvantages for both hypothesis-generating and hypothesis-driven biomarker discovery. Furthermore, as described earlier, platform and assay differences may impact upon the ability to technically replicate findings at the discovery level, and should, therefore, be considered carefully when designing biomarker studies.

## Blood-Based Measures of Aβ and Tau

In the CSF, Aβ_42_ (along with tau and pTau) shows good sensitivity and specificity for classifying AD patients from healthy controls (19). Given the success in developing CSF markers of Aβ and tau as biomarkers it is unsurprising, therefore, that parallel approaches have been attempted in blood.

### Amyloid Beta

Amyloid-beta fragments are produced by β and γ-secretase metabolism of the protein APP. β-secretase cleavage of APP produces sAPPβ and a 99 amino acid membrane bound fragment, which upon subsequent γ-secretase cleavage produces various Aβ species (20). Of these Aβ species, Aβ_40_ is the most abundant, while the highly hydrophobic and insoluble Aβ_42_ is the principal component of amyloid plaques in the AD brain (21), although deposition of insoluble Aβ_40_ in plaques of the AD brain has also been observed (22).

To date, Aβ_42_ and Aβ_40_ are the predominant species that have been investigated in blood, however, as reviewed extensively by others (23, 24), the results of these studies have been somewhat contradictory. To illustrate this, a reduction in plasma levels of Aβ_42_ in mild cognitive impairment (MCI) and AD subjects compared to healthy controls has been shown (25), while no difference between AD and cognitively healthy controls in serum Aβ_42_ has also been reported by others (26).

In terms of disease progression, the results are equally contradictory. An association of decreased plasma Aβ_42_ with more rapid cognitive decline in AD (27), progression from healthy to MCI (28), and conversion from MCI to AD (29) has been shown. Yet an opposite trend has also been reported, including increased Aβ_42_ with conversion from cognitively healthy to MCI (30) and elevated baseline plasma Aβ_42_ in participants who converted to AD versus participants who remained cognitively healthy over a 5-year period (31). Moreover, Mayeux et al. showed that the increase in plasma Aβ_42_ was followed by a decrease in individuals with the onset of AD (31), a pattern that has been mirrored in healthy elderly participants, who demonstrated higher baseline plasma Aβ_42_ followed by greater reductions in plasma Aβ_42_ with cognitive decline (32).

The results of blood Aβ_40_ as an AD biomarker have also been conflicting and are perhaps not as promising as that of Aβ_42_. For example, both increased serum Aβ_40_ (26, 33) and decreased plasma Aβ_40_ (34) have been shown in AD versus healthy controls. Reduced levels of plasma Aβ_40_ have also been associated with more rapid cognitive decline in AD (27), while no change in plasma Aβ_40_ between cognitively stable MCI and MCI to AD converters (29) or association with risk of developing dementia (35) have been observed.

Given the differing results of plasma Aβ_42_ and Aβ_40_ in relation to AD, it is not surprising that studies examining the potential of Aβ_42_/Aβ_40_ in blood as an AD marker have also been conflicting in their results. A number of studies have documented reduced plasma Aβ_42_/Aβ_40_ in association with AD-related parameters, including in MCI and AD subjects compared to healthy controls (25, 28), with progression from MCI to AD compared to cognitively stable MCI (29) and with risk of developing MCI and AD (36). However, increased plasma Aβ_42_/Aβ_40_ has also been related to increased risk of developing AD (37).

Very recently, however, a much larger, prospective, community-based study examined the levels of plasma Aβ_42_ and Aβ_40_ in over 2000 dementia-free individuals, and followed these individuals for dementia/AD over an 8-year period (35). In this study, Chouraki et al., found that lower levels of plasma Aβ_42_ were associated with an increased risk of developing dementia, which given the size of the study may be one of the most promising plasma Aβ results to date.

The conflicting findings of different Aβ studies may perhaps suggest that the utility of plasma Aβ as a marker is quite disease-stage specific, as postulated by Blasko et al. Their findings of a relationship of plasma Aβ with conversion from cognitively healthy to MCI, but not later in the disease course when participants convert from MCI to AD, would indicate that plasma Aβ may be more successful as a marker of pathology at the preclinical stages of disease. This theory would also be in line with why plasma Aβ_42_ appears to perform as a marker of risk for developing dementia over an 8-year period, as documented by Chouraki et al.

### Relationship Between Plasma and CSF Aβ

Given that CSF Aβ is normally cleared in blood (38), it could be hypothesized that a reduction in plasma Aβ_42_ would be observed following the decrease observed in CSF Aβ_42_ in late-onset AD (39). However, a number of studies have actually reported that CSF and plasma levels of Aβ_42_ and Aβ_40_ do not correlate well (40–42). There are several theories that could be proposed to explain this. First, the relationship between CSF and plasma levels of Aβ may only exist at specific stages of the disease, relating to the degree of aggregation of brain amyloid in plaques. Second, it is thought that plasma Aβ may have a causal role in the development of microvascular dysfunction (43) and given the considerable incidence of cerebrovascular pathology in the AD brain (44), it has been proposed that this heterogeneity in pathology could also impact upon the levels of Aβ measured in blood.

### Plasma Aβ and Neuropathology

The relationship between plasma Aβ and brain pathology is also not yet resolved. Levels of Aβ_40_ and Aβ_42_ 1 year prior to post-mortem brain tissue collection were not associated with frontal and temporal necortex Aβ_40_ and Aβ_42_ burden at post mortem (45). However, using PET measures of brain amyloid burden does suggest a relationship between plasma Aβ and brain amyloid load, with an association between reduced plasma Aβ_42_/Aβ_40_ and increased brain amyloid load being shown (28, 46, 47). Moreover, the ratio of the plasma proteins APP669-711 (cleavage product of the amyloid precursor protein) and Aβ_42_ was increased in individuals of high amyloid burden subjects and demonstrated good sensitivity and specificity (93 and 96% respectively) for discrimination of amyloid negative and positive subjects (48).

These findings indicate a potential relationship between plasma Aβ species and the neuropathology of AD, however, given the contradictory results of plasma Aβ as a marker of AD diagnosis and clinical progression, it is clear that further work is required in order to consolidate the findings. As mentioned earlier, potential theories for the variability in the blood Aβ study results have been suggested and include disease-heterogeneity effects upon Aβ levels, and a disease-stage-specific nature of Aβ as a marker, with perhaps Aβ acting as an effective marker of preclinical rather than established disease. While these are valid theories that likely are having an impact, they are not able to explain the full extent of variability between the different Aβ study findings.

Important additional issues that likely contribute to the variability observed between studies are the technical challenges encountered with measuring Aβ. First, Abdullah et al. reported high intra-subject differences in plasma Aβ measures, as assessed by ELISA in two to three separate blood samples retrieved within a 4-week period from each individual (26). This variation in part may be related to the performance of the Aβ assays, with perhaps variation in the measurements being introduced due to lack of sensitivity of these assays. However, it is worth noting that plasma Aβ exhibits a circadian rhythm in its levels (49) and, therefore, in order to use Aβ as a reliable marker, standardization in time of sampling will be required.

Second, it should be noted that many of the studies documented here have assessed plasma Aβ by immunocapture-based approaches, including commercially available and in-house optimized ELISAs (25–27, 29–31, 33, 36, 37, 42, 45, 46), luminex xMAP assays (25, 28, 35, 41, 42), and immunomagnetic reduction (IMR) assays (47). While ELISAs are the gold standard for protein quantification, it is possible that inter-study variation in the results could be introduced by the use of different assays, which use antibodies that recognize different epitopes of Aβ. In this situation, standardization in the assay used across studies so that blood Aβ measures were epitope specific would be advisable.

Another factor to be considered is the technical difficulties of measuring Aβ, which is present at low concentrations in blood and will readily bind other circulating proteins, such as albumin, lipoproteins, and complement factors (50). One way to help overcome this issue might, therefore, be to develop an assay that can measure both free and cell/protein-bound Aβ. This is an approach that has been used to develop the AB test, which quantifies Aβ_40_ and Aβ_42_ peptides that are free in plasma and bound to other proteins in plasma and blood cells^3^. The AB test shows promise for measuring Aβ in an AD-based cohort (51). Chiu and colleagues also report quantification of plasma Aβ by another highly sensitive immunoassay, developed using a technology known as superconducting quantum interference device (SQUID) IMR assay. This technology is based on measuring the magnetic signals produced from nanoparticles, bound to the target molecule of interest and is able to detect plasma Aβ levels as low as 1 pg/ml for Aβ_40_ and 10 pg/ml for Aβ_42_. This is lower than that of standard Aβ_42_ ELISAs, of which the lower limit of detection is generally around 50 pg/ml (52). Furthermore, the SQUID-IMR technology involves the use of iron-nanoparticles and it has been suggested that the iron-chelating effect may inhibit Aβ oligomerization, hence, reducing the issue of non-quantifiable Aβ oligomers (52). While the results of these new assays for Aβ are promising, validation of these assays in further larger and independent cohort studies is required.

### Tau

To date the investigation of plasma tau-based measures and their utility as biomarkers for AD have also been limited, primarily due to tau being an axonal protein and, therefore, of low abundance in blood. Efforts have, therefore, been made to develop more sensitive assays for detection and reliable quantification.

First, Henriksen et al. have reported measurement of specific tau fragments using an ELISA method. These assays quantified specific tau fragments in serum [ADAM10-generated fragment (Tau-A) and caspase-3-generated fragment (Tau-C)] (53, 54). Using this method, measures of serum Tau-A, Tau-C, and the Tau-A/Tau-C ratio were shown to be associated with cognitive change in AD, although no association of the serum tau fragments with CSF tau and pTau were observed (54). A second approach that has been reported for measuring tau utilizes a digital array-based technology (55). This approach involves the isolation and detection of single enzyme molecules using femtolitre-sized reaction chambers, known as single-molecule arrays (SiMOA). This method facilitates the detection of the target at low concentrations by ensuring that the fluorophores are confined to small volumes and, hence, the concentration of fluorescently labeled target is high (56). Using this assay, elevated levels of plasma tau in AD in comparison to controls and MCI were shown, although a considerable overlap in the range of plasma tau across the diagnostic groups was also found (55). Moreover, no correlation between plasma and CSF tau levels were observed (55). Lastly, Chiu and colleagues reported quantification of plasma Tau by SQUID-IMR (as described earlier for detection of plasma Aβ) and showed an increase in plasma tau in MCI and AD, along with an association of plasma tau with clinical measures of cognition and regional brain volume (57). This is all early but promising work, and moving forward, as with blood Aβ measures, further replication of these findings in larger independent cohorts will be crucial for ascertaining the robustness of blood-based tau as an AD-related biomarker.

## Discovery of Blood-Based Biomarkers of AD Using a Case–Control Study Design

Since the blood–brain barrier damage that occurs in AD would facilitate movement of proteins between brain and blood (58), research has also focused upon the detection of other blood-based proteins, in addition to Aβ and tau, which may serve as markers for AD. Using both untargeted and candidate-based proteomic approaches and a case–control study design, a substantial number of proteins related to a diagnosis of AD or MCI have been identified (33, 59–95).

However, a panel of proteins rather than single protein candidates may have greater sensitivity and specificity as a biomarker and may collectively better describe and characterize the disease and its pathology. A number of studies, including from our group, have, therefore, taken an approach of analyzing multivariate signatures for prediction of AD and/or MCI status, and have identified and evaluated different proteins that collectively demonstrate sensitivity and specificity for classifying AD and/or MCI to varying degrees (96–118).

Alzheimer’s disease biomarker studies premised upon a case–control study design have been extensively reviewed by others (119, 120) and as would be expected, many of the candidates identified in these studies can be related to aspects of the disease pathology, for example, having roles in inflammatory and amyloidogenic processes.

These studies comparing established disease to non-disease or prediction of rate of progression in established disease are promising but of more value would be marker sets that detected preclinical or prodromal disease. One design enabling such discovery is the prediction of conversion from MCI by using historical samples from research cohort participants with MCI comparing those who subsequently converted to dementia in a given time-frame to those who did not. One of the first such studies identified an 18 plasma protein signature that not only classified AD from control subjects with 90% accuracy but was also able to predict MCI patients who would convert to AD within 5 years (97). However, replication of the 18 protein biomarker panel in subsequent studies has so far been unsuccessful (103, 121, 122). Yang et al. also demonstrated prediction of MCI conversion to AD with 79% accuracy using a 60 protein biomarker set (123), while we identified a panel of 10 proteins that were shown to strongly associate with both the degree of disease severity and to predict MCI progression to AD with 87% accuracy (124). More recently, Apostolova et al. reported prediction of MCI progression to AD with 73% accuracy by plasma IL-6R combined with clinical measures and *APOE* genotype (125).

Although a number of plasma protein signatures of AD diagnosis, disease severity, and progression have been identified in discovery-based studies, a key concern for the field has been the lack of reproducibility of these results. As yet there has been no single blood-based proteomic signature that can successfully distinguish between AD and MCI and cognitively healthy elderly in a reproducible manner. The reason for such non-reproducibility is unknown. It might be the inherent heterogeneity of the disease and the differences, therefore, between cohort studies. It might also be technical variability, including assay variation and sample collection and curation variation, or it might be that the findings are in fact artifactual and there is no consistent proteomic signature to be found in blood. However, another reason for the failure to replicate might be the intrinsic limitation of case–control studies in a condition with such a long prodrome.

First, it is important to consider the heterogeneity of dementia and the extensive comorbidity and differential environmental exposure in the elderly. As well as multiple dementia conditions being hard to distinguish from each other, the AD group itself can be clinically heterogeneous as can MCI. Moreover, comorbid conditions are common in AD, and might not only alter the blood proteome directly but the associated polypharmacy prevalent in the elderly could also have an impact.

Second, case–control-based studies have inherent limitations when the target of discovery is in prodromal, or, worse, preclinical disease. In the context of AD research, the goal of biomarker discovery is primarily to detect individuals harboring early pathological change but without manifest dementia, as these individuals might be the most likely to respond to disease modifying agents. And yet in case–control studies such individuals will be included in studies not in the “case” group but in the “control” group. Clearly, this study design is at best non-optimal and at worse, destined for failure.

The recent failure of phase III clinical trials of antibody therapies targeting amyloid pathology, in part probably due to the absence of brain amyloid pathology in a considerable proportion of the participants (126, 127), highlights the important role biomarkers predictive of core AD neuropathology could play in recruitment to clinical trials. However, the inevitable screen failures using such approaches would be costly and increase the time to recruitment. Therefore, the development of a minimally invasive blood-based biomarker of AD pathology could have real utility as a first pass or triage marker, to identify potential participants more likely to harbor pathology and to reduce screen failure and, hence, facilitate trials conduct.

## Discovery of Blood-Based Biomarkers of AD Pathology Using an Endophenotype Approach

Endophenotype-based approaches for blood-based biomarker discovery have begun to be implemented and have utilized various AD-related measures to identify blood-based biomarkers reflective of disease activity and pathology, including at the preclinical stages. These studies have included endophenotypes defined by measures such as brain atrophy (structural MRI), rate of cognitive decline, and brain amyloid β burden (Pittsburgh B (PiB) PET brain imaging), with change in PiB PET amyloid burden being the earliest event of these in the disease course. These studies have identified a number of different potential proteomic biomarkers (Tables 2 and 3).

**Table 2:** **Summary of the significant findings of studies examining plasma protein markers of brain atrophy and rate of cognitive decline**.

Proteins	Outcome variables (subjects)	Analytical platform	Study
**Endophenotype: structural MRI measures of brain atrophy**			
C3, FGG, albumin, CFI, clusterin, A1M and SAP	Hippocampal atrophy (AD and MCI)	2DGE LC-MS/MS	(128)
C3, C3a, CFI, FGG, and A1M	Whole brain volume (AD)	ELISA and western blots	(129)
ApoB/ApoA1^a^, ApoC3^b^, ApoE^b^, and Clusterin^b,c^	Hippocampal volume^a^, gray matter volume^b^, and white matter volume^c^ (MCI and non-demented elderly)	Luminex xMAP (Myriad RBM)	(130)
Clusterin	Rate of brain atrophy (multiple brain regions in MCI)	ELISA	(131)
IL-1ra^d^ IL-6^d^, IL-10^d^, IL-13^e^, and TNF-α^f^	Ventricular volume^d^, entorhinal cortex volume^e^, and whole brain volume^f^ (AD)	Luminex xMAP	(132)
MIP1α, IGFBP2, CgA, and cortisol	SPARE-AD measures of brain atrophy (AD, MCI, and non-demented elderly)	Luminex xMAP (Myriad RBM)	(133)
RANTES^g^, NSE^g,h^, TTR^g,h^, clusterin^g^, A1AT^h^, ApoC3^h^, ApoA1^h^, ApoE^h^, BDNF?^h^ and Aβ_40_^h^	Atrophy in multiple brain regions (MCI^g^ and AD^h^)	Luminex xMAP	(124)
PPY, fetuin B, PSA-ACT, and ChkT	Entorhinal cortex and hippocampal volume (AD, MCI, and non-demented elderly)	SOMAscan	(134)
ApoE	Hippocampal volume (MCI, non demented elderly)	Luminex xMAP (Myriad RBM)	(135)
**Endophenotype: rate of cognitive decline**			
C4a, C8, clusterin, ApoA1, and TTR	Rate of cognitive decline (AD)	2DGE LC-MS/MS	(128)
ApoA1, ApoA2, ApoH, and ApoB/ApoA1 ratio	Risk of cognitive decline (non-demented elderly)	Luminex xMAP (Myriad RBM	(130)
TTR	Rate of cognitive decline (AD)	ELISA	(136)
IL-4, IL-10, G-CSF, IL-2, IFN-γ, and PDGF	Rate of cognitive decline (AD)	Luminex xMAP	(132)
NCAM, sRAGE, and ICAM	Rate of cognitive decline (AD)	Luminex xMAP	(124)
Clusterin and NAP2	Rate of cognitive decline (AD)	SOMAscan	(134)

*C3, complement C3; FGG, γ-fibrinogen; CF1, complement factor-I; A1M, α-1-microglobulin; SAP, serum amyloid-P; C3a, complement C3a; ApoB, apolipoprotein B; ApoA1, apolipoprotein A1; ApoC3, apolipoprotein C3; ApoE, apolipoprotein E; ApoC4, apolipoprotein C4; IL-1ra, interleukin 1 receptor antagonist; IL-6, interleukin-6; IL-10, interleukin-10; IL-13, interleukin-13; TNF-α, tumor necrosis factor alpha; MIP1α, macrophage inhibitory protein 1α; IGFBP2, insulin-like growth factor binding protein 2; CgA, chromogranin A; RANTES, regulated on activation normal T cell expressed and secreted; NSE, neuron-specific enolase; TTR, transthyretin; A1AT, alpha 1 antitrypsin; BDNF, brain derived neurotrophic factor; Aβ_40_, amyloid beta 1-40; PPY, pancreatic polypeptide; PSA-ACT, prostate-specific antigen complexed to α1-antichymotrypsin; Chk2, serine/threonine-protein kinase Chk2; C4a, complement component C4a; C8, complement C8; ApoA2, apolipoprotein A-2; ApoH, apolipoprotein-H; IL-4, interleukin-4; G-CSF, granulocyte-colony-stimulating factor; IL-2, interleukin-2; IFN-γ, interferon-gamma; PDGF, platelet-derived growth factor; NCAM, neural cell adhesion molecule; sRAGE, soluble receptor for advanced glycation end products; ICAM, intercellular adhesion molecule; NAP2, nucleosome assembly protein 2*.

**Table 3:** **Summary of the significant findings of studies examining plasma protein markers of PET amyloid**.

Protein(s)	Outcome variable (subjects)	Analytical platform	Study
Clusterin	PiB PET amyloid (non-demented elderly)	ELISA	(128)
ApoE, C3, albumin, plasminogen, haptoglobin and IgG C chain region	PiB PET amyloid (non-demented elderly)	2DGE LC-MS/MS	(137)
C-peptide, fibrinogen, A1AT, PPY, C3, vitronectin, cortisol, AXL receptor kinase, IL-3, IL-13, MMP9, ApoE, and IgE (this panel of proteins combined with covariates predicts amyloid positive subjects with 92 and 55% sensitivity and specificity, respectively)	PiB PET amyloid (AD, MCI, and non-demented elderly)	Luminex xMAP (Myriad RBM)	(138)
Aβ1–42, CXCL-13, IL-17, IgM-1, PPY, and VCAM-1 (this panel of proteins with age, *APOE* genotype, and CDR sum of boxes predicts NAB with 79 and 76% sensitivity and specificity, respectively)	PiB PET amyloid (AD, MCI, and non-demented elderly)	Luminex xMAP (Myriad RBM)	(139)
A2M, CFHR1, and FGG. (FGG in combination with age predicts NAB with 59 and 78% sensitivity and specificity, respectively)	PiB PET amyloid (AD, MCI, and non-demented elderly)	TMT LC-MS/MS	(140)
IL-6R, ApoE, and clusterin (in combination with clinical measures: trails B, AVLT, MMSE, education, *APOE* genotype and mean hippocampal volume predicts NAB with 79 and 83% sensitivity and specificity)	CSF Aβ and PiB PET amyloid (MCI)	Luminex xMAP (Myriad RBM)	(125)
BDNF	PiB PET amyloid (AD, MCI and non-demented elderly)	Luminex xMAP (Myriad RBM)	(141)
PPY and IgM*	PiB PET amyloid (AD, MCI, and non-demented elderly, *non-demented elderly only)	SOMAscan	(142)

*ApoE, apolipoprotein E; C3, complement C3; A1AT, alpha 1 antitrypsin; PPY, pancreatic polypeptide; IL-3, interleukin-3; IL-13, interleukin-13; MMP9, matrix metalloproteinase-9 total; ApoE, apolipoprotein E; IgE, immunoglobulin E; CKCL-13, chemokine ligand 13; IL-17, interleukin-17; IgM-1, immunoglobulin M; VCAM-1, vascular cell adhesion protein; A2M, alpha 2 macroglobulin; CFHR1, CFH-related protein 1; FGG, fibrinogen gamma chain; NAB, neocortical amyloid burden*.

*non demented elderly only.

### Blood-Based Biomarkers of Brain Atrophy and Rate of Cognitive Decline

We began by focusing on endophenotype approaches using mostly the AddNeuroMed, a European multicentre study (143) and the neuroimaging substudy of the Baltimore Longitudinal Study for Aging (BLSA) (144). Two key pathology endophenotypes were employed; structural neuroimaging of atrophy as a proxy measure of *in vivo* pathology and rate of clinical progression (Table 2), which was calculated based on retrospective and prospective measures of cognitive decline.

In 2010, we published a study that utilized a 2DGE-MS/MS-based approach to discover plasma protein markers of both of these outcome variables in AD (128). This work identified seven proteins (complement C3, γ-fibrinogen, serum albumin, complement factor-I, clusterin, α-1-microglobulin, and serum amyloid-P) that were able to explain 34% of the variance in hippocampal volume in MCI and AD, and five proteins (complement component C4a, complement C8, clusterin, ApoA1, and transthyretin) that were able to discriminate fast from slow progressing AD groups. These proteins were then selected for replication studies, including in an independent AD/MCI/control-based cohort, using an orthogonal immunoassay-based approach. In this study, we replicated the association of complement C3, complement factor-I, γ-fibrinogen and α-1-microglobulin with brain atrophy, and along with complement C3a, these five proteins were able to explain 35% of whole brain volume in AD (129). In a separate study, we also replicated the association of transthyretin with an increased rate of cognitive decline in AD (136).

However, the most promising candidate marker identified in this discovery study was the protein clusterin, which associated with both hippocampal atrophy and clinical progression (128). We also showed in this same study but in an independent (AD/MCI/control) cohort, an association of clusterin with cognitive measures and with brain atrophy, specifically in the entorhinal cortex and with PiB PET measures of fibrillary amyloid burden in the entorhinal cortex of a non-demented elderly cohort (128). While very recently increased plasma clusterin levels have been associated with increased risk of conversion to AD and rate of cognitive decline in an independent study (145). These findings indicate that changes in plasma clusterin may be an early event in the disease course, which occurs with amyloid deposition but prior (or without) onset of clinical symptoms. Moreover, in this same study, we demonstrated colocalization of clusterin with Aβ in plaques in the brains of a transgenic mouse model of AD (TASTPM) (128), thus, adding further support to the theory that clusterin may be implicated in amyloid formation and clearance (146).

Adding weight to our hypothesis that changes in plasma clusterin were an early event, increased levels of plasma clusterin in association with slower rates of brain atrophy in MCI were demonstrated (131). However, to the contrary, Song et al. demonstrated an association of increased plasma clusterin with reduced white matter volume in MCI/cognitively healthy elderly over a 2-year period (130). These findings are somewhat contradictory, and could be explained in part by the evidence for clusterin having both neuroprotective and pro-amyloidegenic properties, dependent on its concentration relative to Aβ. Clusterin is implicated in Aβ aggregation and clearance (146–151) and at high concentrations, clusterin binds Aβ, thus, preventing its aggregation. Yet when Aβ levels are high, clusterin instead is incorporated with amyloid in insoluble aggregates (148). Furthermore, clusterin possesses neurotoxic properties, as demonstrated by its involvement in non-canonical wnt signaling (the wnt–PCP–JNK pathway), which mediates Aβ toxicity (152). It could, therefore, be postulated that clusterin is playing different roles in these studies that demonstrate opposing relationships of plasma clusterin with brain atrophy. Nonetheless, these studies add further evidence for the role of clusterin in AD pathology. It is also worth noting that evidence for clusterin being implicated in AD pathology has also been provided on the genetic level, with an association of the variant rs11136000 in the clusterin gene with AD risk (153, 154), increased rates of cognitive decline at the pre-symptomatic stages of the disease (155) and brain volume and structure (volumetric expansion and lateral ventricle surface morphology) in AD, MCI, and elderly control subjects (156).

To date, clusterin is likely to be the most promising potential biomarker of AD-related phenotypes that we have identified in our studies, as supported by an association on the proteomic level with both clinical and neuroimaging measures of AD pathology, on the genetic level with AD risk and on a mechanistic level with amyloid function and processing.

Following the identification of clusterin using a dual endophenotype-based approach founded upon both brain atrophy and cognitive decline measures, we sought to extend this approach further to find biomarkers of these endophenotypes using different proteomic methods, which may be more sensitive for detection of alternative groups of proteins. One such study was reported by Sattlecker et al. who utilized the SOMAscan technology for plasma protein biomarker discovery in a cohort of AD, MCI, and controls. The strongest findings of this study included an association of clusterin with cognitive decline, replicating the findings of Thambisetty et al. (128), along with an association of fetuin B and pancreatic polypeptide with brain atrophy, and an association of pancreatic polypeptide and PSA-ACT with a diagnosis of AD (134).

In addition to hypothesis generating discovery approaches, targeted hypothesis-driven approaches have also been successful in identifying potential biomarkers of brain atrophy and cognitive decline. For example, the apolipoprotein family is widely implicated in neurodegeneration (157, 158) and in a targeted study, Song et al. showed a negative correlation of plasma clusterin and ApoE with gray matter volume and an association of ApoA1, ApoA2, ApoH, and the ApoB/ApoA1 ratio with risk of cognitive decline in cognitively normal individuals (130). As these proteins are associated with pathology-related outcomes at the preclinical stage of disease, this would suggest that the apolipoproteins may be markers in an early phase of the disease pathogenesis. More recently, Teng et al. also showed an association of plasma ApoE levels with hippocampal volume in a cohort of AD, MCI, and control included in the Alzheimer’s disease neuroimaging initiative (ADNI) cohort (135).

We have also taken a targeted approach to examine the biomarker potential of inflammatory proteins (132), given the evidence for an inflammatory component in AD pathology (159, 160). We observed five proteins that were associated with brain atrophy measures (IL-1ra, IL-6, IL-10, TNF-α, and IL-13) and six proteins that were associated with rate of cognitive decline in AD (IL-4, IL-10, G-CSF, IL-2, IFN-γ, and PDGF) (132). Of note was the association of IL-10 with both brain atrophy and rate of cognitive decline, adding further confidence to the finding of its association with AD-related endophenotypes (132). Toledo et al. also published findings of inflammatory proteins (macrophage inflammatory protein 1 alpha, chromogranin A) along with proteins implicated in the stress response (cortisol) and insulin response (insulin-like growth factor binding protein 2) as markers of brain atrophy (133).

Following the identification of various plasma proteins related to AD and proxy measures of disease activity (neuroimaging measured of brain atrophy and clinical measures of cognitive decline), we next sought to validate the most promising and disease-relevant protein markers. To do this, we used multiplex bead assays to measure candidate proteins in a larger (*N* > 1000) cohort of AD/MCI/control participants (124). Interestingly, we found that different sets of proteins were associated with brain atrophy in MCI compared to AD, indicating that these markers are disease-phase specific, and the strongest associations with brain atrophy were observed for clusterin in the MCI group and ApoE in the AD group (124). Furthermore, we identified three proteins NCAM, sRAGE, and ICAM as being associated with rate of cognitive decline and we, therefore, hypothesized that these markers may be predictive of conversion from MCI to AD. When we tested this, we found that there were a panel of 10 proteins (transthyretin, clusterin, cystatin C, A1AcidG, ICAM1, complement component C4, PEDF, A1AT, RANTES, and ApoC3) along with *APOE* genotype, which were able to predict MCI conversion to AD with 87% accuracy, 85% sensitivity, and 88% specificity, as described earlier (124).

### Blood-Based Biomarkers of Brain Amyloid Burden

Blood-based biomarkers of neocortical Aβ (extracellular β-amyloid) burden (NAB) as measured by PET brain imaging have also been sought (Table 3). These studies have used the Australian Imaging, Biomarker and Lifestyle Flagship Study of Ageing (AIBL) (161), the ADNI^4^, and the BLSA (144) for the purpose of finding plasma proteomic markers of brain amyloid burden.

The first study we carried out used the BLSA study to discover plasma proteins that were associated with NAB in non-demented elderly individuals (137). Using a 2DGE-MS/MS-based approach, this study identified six proteins (ApoE, Complement C3, Albumin, Plasminogen, Haptoglobin and IgG C chain region) that discriminated “high” from “low” PiB PET brain amyloid burden subjects in discovery-based studies, and a further association of ApoE with amyloid burden in the medial temporal lobe in an independent validation study (137).

Following this, we carried out a separate study to examine the association of plasma proteins with NAB in AD, MCI, and control subjects included in the ADNI^5^ (138). Plasma proteins were measured by the Myriad Rules-Based Medicine (RBM) panel using commercially available multiplexed luminex assays. This work identified 13 plasma proteins (c-peptide, fibrinogen, A1AT, pancreatic polypeptide, complement C3, vitronectin, cortisol, AXL receptor kinase, IL-3, IL-13, matrix metalloproteinase-9 total, ApoE, and IgE), which in combination with covariates were able to discriminate PiB-positive from PiB-negative individuals with 92 and 55% sensitivity and specificity, respectively (138).

Shortly after this, Burnham et al. published a study that again utilized the RBM panel for identifying plasma proteins predictive of NAB in an AD, MCI, and control-based population; however, this study utilized the AIBL study for discovery, followed by validation of potential biomarkers of NAB in the ADNI (139). In summary, Burnham et al. identified six plasma proteins (Aβ_42_, chemokine ligand 13, IL-17, IgM-1, pancreatic polypeptide and VCAM-1) that contributed to a biomarker signature that was able to predict NAB with 79 and 76% sensitivity and specificity in the ADNI-based validation cohort (139).

More recently, a study carried out in an ADNI-based MCI cohort revealed that plasma IL-6 receptor, clusterin, and ApoE levels coupled with a number of clinical and demographic measures, *APOE* genotype and mean hippocampal volume, achieved 79 and 83% sensitivity and specificity for prediction of NAB (125). Hwang et al. also reported an association of reduced plasma BDNF levels with increased regional measures of NAB in an ADNI cohort (141).

We also recently reported the results of an LC-MS/MS-based approach for the discovery of plasma protein biomarkers of NAB in AD, MCI, and healthy controls enrolled in the AIBL study (140). Using this approach, a number of plasma proteins were shown to be significantly associated with NAB, including A2M, CFH-related protein 1, and γ-fibrinogen. Moreover, the association of γ-fibrinogen in combination with age was found to predict NAB with 59 and 78% sensitivity and specificity, respectively (140).

Although the exact protein biomarker panels identified by these studies for prediction of NAB differs between the studies, it is of note that there are some commonalities in the proteins included in these biomarker panels, including ApoE (125, 137, 138), complement C3 (137, 138), and pancreatic polypeptide (138, 139). A recent study, therefore, sought to replicate these findings in an independent cohort of AD, MCI, and control subjects in the AIBL study (142). This work replicated an association of two proteins with NAB; pancreatic polypeptide across the cohort of AD, MCI, and cognitively healthy elderly, and IgM in the cognitively healthy elderly group, while the association of the other protein candidates with NAB was not replicated (142). This lack of replication between studies is disappointing; however, it is quite possible that this could be in part due to technical platform differences, as the discovery studies used both MS (137, 140) and immunocapture-based approaches (125, 138, 139, 141), while replication was sought using the SOMAscan platform (142). As mentioned earlier, platform and assay differences may provide differing quantitative proteomic results, given that there are key differences in the nature of the protein being measured by these techniques. MS approaches measure denatured protein in a peptide-specific manner, while immunocapture-based assays use antibodies for epitope-specific native protein measures. The SOMAscan platform also measures native protein, but by binding of an aptamer to a tertiary structure-specific epitope. Therefore, differences in the region and confirmation of the protein target being measured by these different techniques may result in varying quantitative results.

These various studies utilizing an AD pathology endophenotype-based approach for biomarker discovery show promise in identifying biomarkers reflective of core AD pathology and disease activity. However, it is important to note that there are some issues surrounding the approach of predicating blood-based biomarker discovery on PET amyloid measures. First, PiB PET detects insoluble fibrillary but not insoluble oligomeric Aβ, which are known to possess neurotoxic and synaptotoxic properties (162). Therefore, blood-based biomarkers of PiB PET amyloid may not be the most relevant markers of brain amyloid pathology. Second, it is possible that the relationship of plasma proteins with PiB PET amyloid measures could be specific to the technical aspects of the PET imaging technique used. For example, variability in the amyloid measure could be introduced by the use of alternative radiotracers or alternative methods of PET data analysis.

Therefore, in order to assess the reproducibility and robustness of plasma proteins biomarkers of amyloid (as indicated by PiB PET), it will be essential to perform replication and validation studies examining their association with brain amyloid burden (1) in larger independent cohorts, (2) using orthogonal technical platforms for biomarker quantification, and (3) using alternative measures indicative of amyloid (for example, alternative PET amyloid radiotracers and CSF Aβ).

### Other Potential Endophenotype Approaches

While endophenotype-based designs founded upon rates of cognitive decline, brain atrophy, and brain amyloid burden show promise, there are further measures of AD and other aspects of core AD neuropathology that warrant investigation as potential endophenotypes for biomarker discovery. FDG-PET measures cerebral metabolic glucose utilization rate and serves as an indicator of synaptic activity, neuronal function, and neuronal metabolic activity (163). FDG-PET has been reported to have an average diagnostic accuracy of 93% (96% sensitivity and 90% specificity) for differentiating AD from cognitively healthy elderly subjects (164), and can discriminate between different dementia-types with around 94% accuracy (165). Using FDG-PET as an endophenotype of pathology for blood-based biomarker discovery could, therefore, aid in the development of biomarkers relating to synaptic and neuronal function, and the prodromal stage of disease, given that hypometabolism is known to occur in amnestic MCI (165, 166). Moreover, glucose metabolism is thought to be more closely associated with certain memory, language, and visuospatial clinical variants of AD than measures of Aβ deposition and so plasma protein biomarkers of FDG-PET cerebral glucose metabolism could be of utility in detecting these clinical aspects of the disease (167).

With the development of tau imaging comes the opportunity to investigate blood-based biomarkers related specifically to brain tau pathology, which could obviously be of potential utility beyond AD and for tauopathies such as fronto-temporal dementia. The development of tau imaging has been challenging due to the deposition of tau protein being intracellular, which impacts upon radiotracer binding and image contrast (168). However, current research to develop various tau brain imaging tracers is underway, including the tracers 18F-THK523, [F-18]-T807, and [F-18]-T808 (169–171). PET imaging of tau could, therefore, provide another endophenotype parameter for the design of studies that seek to uncover peripheral proteomic biomarkers relating specifically to tau pathology in the brain.

Moreover, other types of biomarkers detectable in the blood show promise as potential markers of AD, including, for example, metabolomic (172–175) and transcriptomic-based markers (176, 177). Further research to examine how these markers may be related to pathology endophenotypes and the potential of combining these markers as a multimodal signature of AD pathology will be important.

## Conclusion

Much research has sought blood-based proteomic biomarkers that may have diagnostic utility in discriminating AD cases from control, with limited success in identifying a reproducible signature of diagnostic or trials utility.

An alternative approach, which we have increasingly employed is using surrogates for disease activity – endophenotypes – such as cerebral atrophy imaging or molecular markers of amyloid pathology and rate of decline. Such an approach yields different but overlapping panels of markers. It is, therefore, possible that such markers predicated on pathological processes might be more reproducible and ultimately of more utility in diagnostic, prognostic, predictive, and other utilities especially in the context of clinical trials.

However, it seems intrinsically unlikely to us that a blood-based biomarker would replace relatively specific and reliable markers such as molecular markers in CSF or PET imaging markers that are more proximal to the disease state. Rather, we predict that blood-based biomarkers might be less specific but possibly more sensitive and certainly more readily conducted repeatedly in the context of large-scale, community-based studies and where repeated measures to track change is required. This then raises the prospect of what might be termed the biomarker funnel, a series of tests and investigations starting with the minimally invasive, highly sensitive, poorly specific marker set leading toward a technologically demanding or invasive test that is highly specific. This would be a blood test triage or selection process for CSF or PET tests in effect. Such a funnel is commonplace in medicine – fasting glucose before a glucose tolerance test, mammography before biopsy are examples, but there are many others. A biomarker funnel with blood-based markers as an early step toward a pathological diagnosis in life would be a very substantial step forward and maybe an essential step before clinical trials can be both effective and achievable.

## Author Contributions

AB, SW, and SL all contributed to the design of the review and interpretation of the studies included within it. AB, SW, and SL all contributed to the drafting and revision of the content and provided final approval of this version to be published.

## Conflict of Interest Statement

Simon Lovestone is named as an inventor on biomarker intellectual property patent protected by Proteome Sciences and Kings College London. Alison L. Baird and Sarah Westwood have no conflict of interest to declare.

## Footnotes

^1^http://www.ncbi.nlm.nih.gov/pubmed

^2^www.somalogic.com

^3^www.araclon.com

^4^www.adni-info.org

^5^adni.loni.ucla.edu
